# Investigating immune responses to parasites using transgenesis

**DOI:** 10.1186/s13071-019-3550-4

**Published:** 2019-06-15

**Authors:** Mebrahtu G. Tedla, Alison L. Every, Jean-Pierre Y. Scheerlinck

**Affiliations:** 1 Centre for Animal Biotechnology, Faculty of Veterinary and Agricultural Sciences, The University of Melbourne, Melbourne, VIC 3010 Australia; 2 Present Address: College of Science, Health and Engineering, La Trobe University, Melbourne, VIC 3086 Australia

**Keywords:** Transgenesis, Parasites, Immune response, *In vivo*

## Abstract

Parasites comprise diverse and complex organisms, which substantially impact human and animal health. Most parasites have complex life-cycles, and by virtue of co-evolution have developed multifaceted, often life-cycle stage-specific relationships with the immune system of their hosts. The complexity in the biology of many parasites often limits our knowledge of parasite-specific immune responses, to *in vitro* studies only. The relatively recent development of methods to stably manipulate the genetic make-up of many parasites has allowed a better understanding of host-parasite interactions, particularly *in vivo*. In this regard, the use of transgenic parasites can facilitate the study of immunomodulatory mechanisms under *in vivo* conditions. Therefore, in this review, we specifically highlighted the current developments in the use of transgenic parasites to unravel the host’s immune response to different life-cycle stages of some key parasite species such as *Leishmania*, *Schistosoma*, *Toxoplasma*, *Plasmodium* and *Trypanosome* and to some degree, the use of transgenic nematode parasites is also briefly discussed.

## Background

Today, despite available treatments, more than 2 billion people are suffering from chronic infections with parasites, resulting in considerable morbidity [1]. Chronic infections develop because these parasites escape destruction by the immune system, arguably helped by their complex biology [2]. Parasitic infections and the host’s immune responses are the result of dynamic co-evolution of the host and the parasite’s complex life-cycle with each life stage resulting in a different interaction with the immune system [3, 4]. Despite significant progress studying the biology of these parasites, there is still incomplete understanding of the interaction between the host’s immune response and these parasites [5].

Recent progress has been made in the development of transgenic parasites allowing for the structural and functional analysis of specific gene products [6], by for example, knocking-in or knocking-out target genes followed by functional analysis, *in vivo* [7]. For genetic manipulation of protozoan parasites (*Toxoplasma*, *Plasmodium* and *Leishmania*), robust systems were developed based on classical molecular biology methods such as microinjection, chemical transfection and electroporation, resulting in early advances in the understanding of the interactions between the immune system and these parasites. In contrast, for multicellular parasites these methods were not optimal and therefore, these parasites are now manipulated using transduction based on retroviral vectors.

Another breakthrough was the discovery that parasites possess the RNA-dependent gene silencing machinery and hence RNA interference (RNAi) has been applied to specifically downregulate genes, as opposed to producing a completely knocking-out of the gene of interest. For multicellular organisms such as *Schistosoma mansoni* lentivirus transduction was necessary to allow a highly efficient introduction of the foreign DNA [8]. Despite such progress, there are still limitations in gene manipulation approaches in multicellular parasites due to their diverse cellular elements and tissues, and complex life-cycles [9].

This review highlights the contributions made by studies on transgenic parasites to the understanding of host-parasite interactions and other applications of genetic manipulations in parasites were specifically omitted.

## Methods

**Fig. 1:**
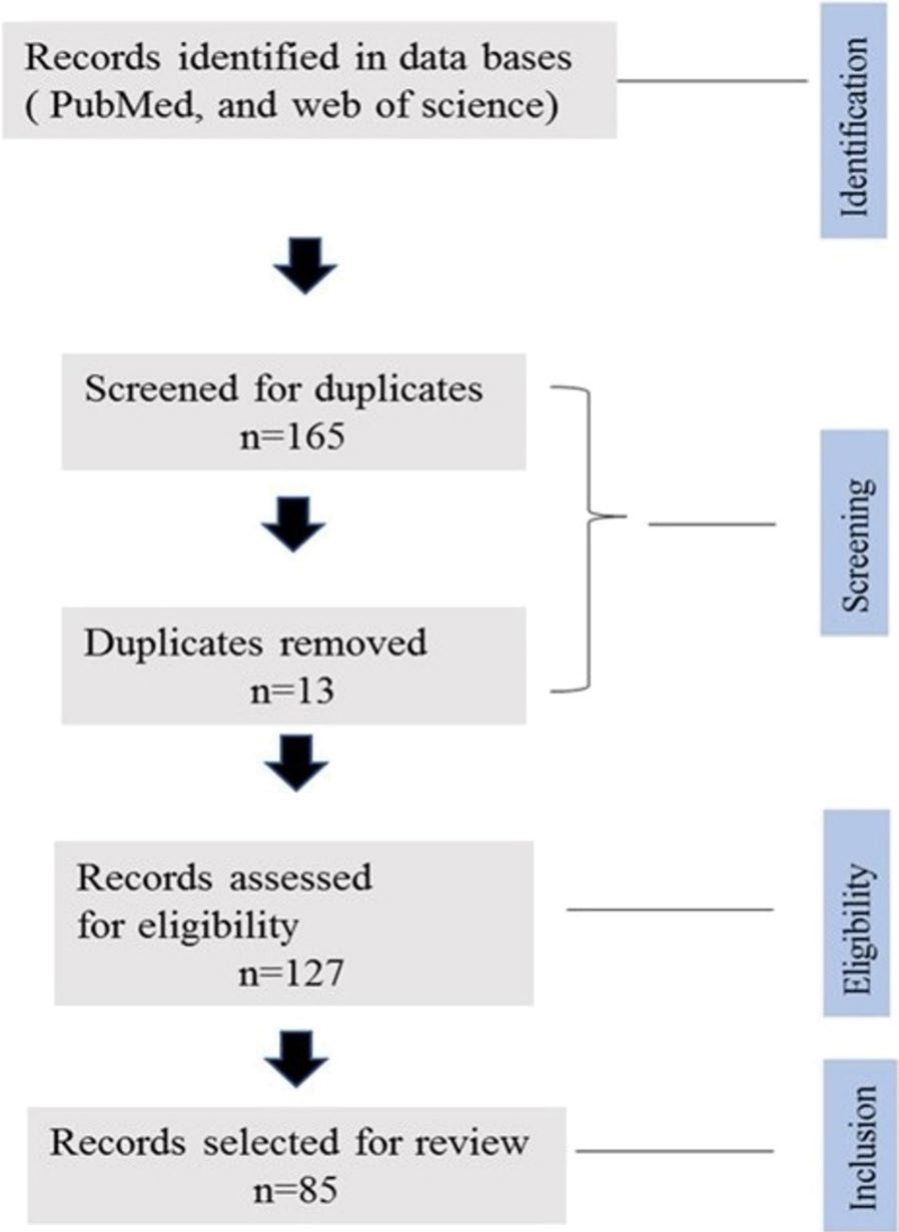
A flow diagram for the identification and screening of research articles for the current review

The databases PubMed and Web of Science were searched using keywords, including “transgenesis”, “parasite” and “immune response”. The search was conducted during the period of 2017 and 2018 and all the relevant scientific publications were screened following the procedure illustrated in the diagram below (Fig. 1). To meet the inclusion and exclusion criteria, only papers containing information on transgenic parasites and immune response study were included during the screen and non-published data or theses were not included for this study purpose.

### Expression of model antigens in parasites

One way of demonstrating protective cell-mediated immune responses is with the help of parasites transfected with putative protective antigens, followed by cell-transfer experiments to confirm the protective immune response. One special case of this approach consists of using antigens for which T cell receptor (TCR) transgenic mice have been generated [10]. These mice express the TCR specific for a peptide present in the model antigen presented in association with either MHC class II or I. For example, OT-II mouse—on a C57Bl/6 mouse background—express a TCR, which specifically recognises a peptide from chicken ovalbumin (OVA, amino acids 323–339) in association with MHC class II. In contrast, OT-I mouse express a transgene encoding a TCR specific for a short peptide fragment of OVA (amino acids 257–264) presented by MHC class I [11]. These mice can be used experimentally as a source of naïve T cells with a known specificity [12] and, hence, offer interesting tools to study T cell responses to these parasites. For example, transgenic *Plasmodium berghei* parasites were generated to express MHC class I- and II-restricted T cell peptide epitopes [13], recognised by TCR transgenic (HNT, DO11.10, and B6) mice, which were then used to elucidate the role of T cells in protective immunity to blood stage parasites. To achieve this, a T cell polytope was generated by artificially linking several T cell epitopes together including (i) MHC-I and II restricted epitopes from OVA, (ii) MHC-I restricted epitope from glycoprotein B of HSV-1, and (iii) MHC-I and II restricted epitopes from influenza virus strain PR8 hemagglutinin [13]. Blood stage of parasites transduced with this polytope were able to induce not only antigen-specific CD4^+^ T cells but, more unexpectedly, also antigen-specific CD8^+^ T cell responses *via* cross-presentation by CD8α^+^ subsets of dendritic cells (DCs). While these antigen-specific CD8^+^ T cells did not contribute to protection against the blood stage of *Plasmodium* infection, they played a key role in the initiation of cerebral malaria (CM) and hence are important in the pathology of this infection [13]. This unexpected finding would have been very difficult to discover without the use of transgenic parasites, hereby demonstrating the usefulness of this technique for the analysis of immune responses to parasites.

Model antigens (i.e. antigens that are not naturally occurring in any pathogen), such as OVA, are frequently expressed in pathogens to investigate the major determinants and pathways influencing T cell responses. In contrast to pathogen-antigens, model antigens have never been subjected to evolutionary pressure and as a result they are unlikely to have been selected to induce biased immune responses. Thus, studying the immune response to these antigens not only allows for the use of many available reagents, but also facilitates the isolation of effects due to the antigen from these due to the pathogen. However, these model antigens are also artificial and therefore may not necessarily reflect the immune responses against pathogen antigens. Nevertheless, they represent useful tools in furthering our understanding of immune responses associated with parasites.

Other examples of the use of model antigens are studies showing that OT-II CD4^+^ and OT-I CD8^+^ T cells specifically recognize epitopes of OVA in *Toxoplasma gondii* including strains expressing OVA such as type I strain (expressing RH-OVA) and type II strains (expressing Pru-OVA). By injecting these transgenic parasites into mice, Pru-OVA can activate the DCs to produce cytokines within the draining lymph node and a large population of endogenous OVA-specific CD8^+^ T cells can be generated. Mice infected with RH-OVA have fewer DCs and OVA-specific CD8^+^ T cells [14]. The expression of model antigens in parasites allows not only the study of the major parasite determinants influencing immune responses, but also the exploration of the effects of the antigens’ subcellular localization on the induction of T cell response.

The expression of model antigens in different part of the parasite using transgenesis have been frequently used to determine the effect of antigen localisation on the host’s immune recognition. For example, *P. berghei* have been developed expressing recombinant proteins either in the cytosol or the parasitophorous vacuole membrane (PVM) to assess whether the sub-cellular location can influence T cell responses. OVA and mCherry conjugated with *P. berghei* heat-shock proteins such as HSP-70 were expressed in the cytoplasm. However, OVA gene sequence conjugated with the HEP17 (EXP1) promoter showed a low level of expression in the PVM [15]. Although both transgenic *P. berghei* lines induce OVA-specific CD8^+^ and CD4^+^ T cell responses, PVM-expressed OVA-HEP17 (EXP1) induced higher proliferation of OT-II and OT-I T cells than cytoplasma-expressed OVA-mCherry-HSP70, suggesting that antigen localisation within the parasite affects T cell recognition. Unconjugated OVA is not expressed in the cytoplasm of the parasite and more importantly here, the location and the degree of expression in the parasite also influence T cell responses [15]. The recognition of transgenic cytoplasmic OVA in *Plasmodium* by CD8^+^ T cells during different life-cycle stages of infection was also described [16]. However, those parasites were unable to induce a CD4^+^ T cell response *in vivo* [17]. A possible explanation for this dichotomy is that the localisation of the antigen in parasites drives the antigen processing pathways used in antigen presenting cells [18]. Furthermore, OVA expressed by blood stage *P. berghei* showed cross-presentation of the antigen by CD8α^+^ antigen presenting cells in association with MHC class I and II inducing both CD8^+^ and CD4^+^ T cells [15].

**Fig. 2:**
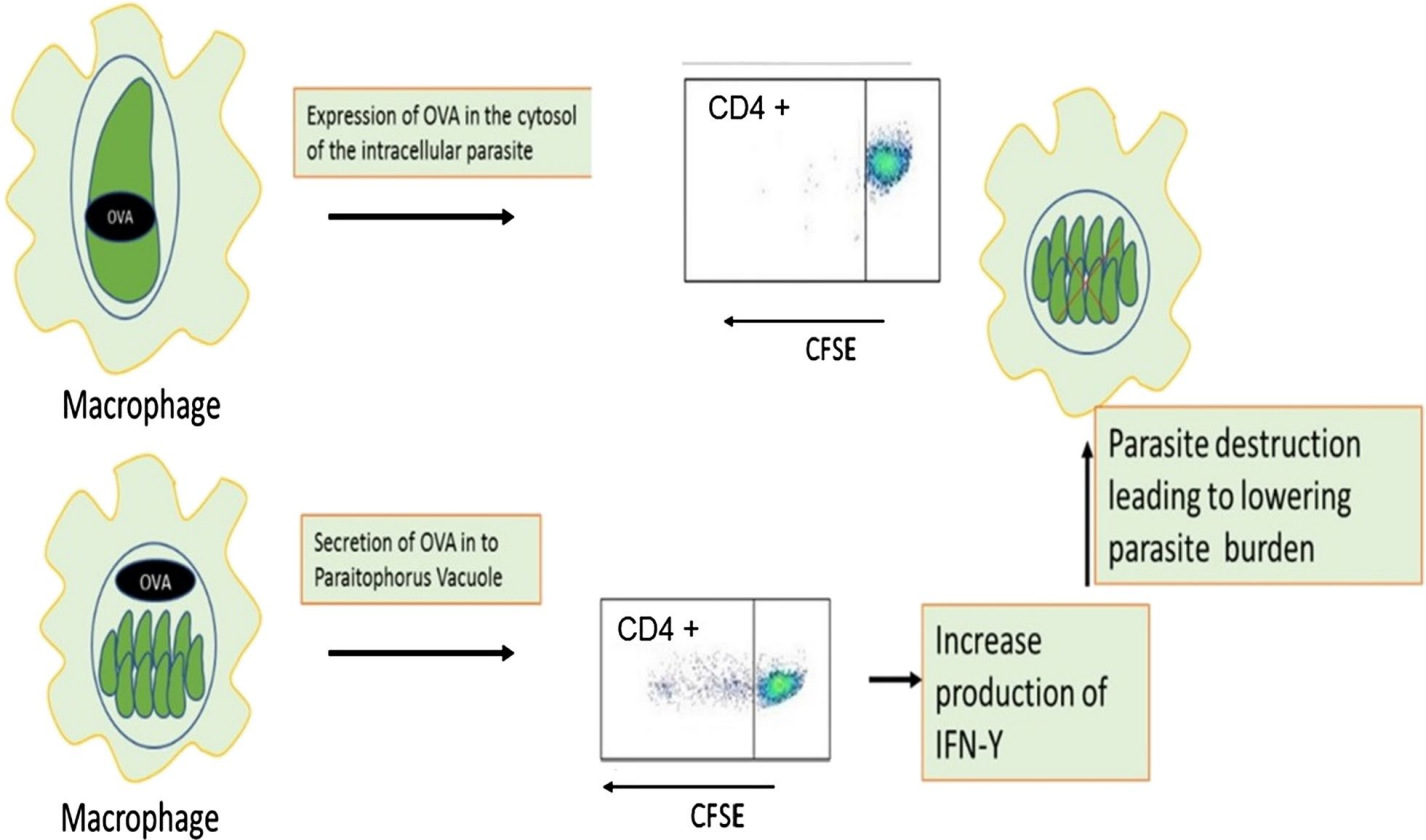
The site of OVA expression inside the transgenic parasite has influenced the cellular immune responses in which cytosolic OVA leads to stimulation of CD4^+^ T cells and IFN-γ production. To confirm whether OVA expressing transgenic *T. gondii* can induce T cell proliferation, CD4^+^ T cells were labelled with CFSE and the *in vitro* response of the cells showed the cytosolic OVA failed to show any T cell response, but OVA expressed at the parasitophorous vacuole induced T cell proliferation [25]

*Plasmodium* spp. are not the only parasites for which the location of the antigen affects recognition by T cells. Indeed, *Toxoplasma gondii* has been engineered to express recombinant OVA either in the cytosol or to be secreted into the parasitophorous vacuole [19]. These studies demonstrated that only the secreted form of OVA, can stimulate CD4^+^ T cells and induce IFN-γ production (Fig. 2). Similarly, LacZ-expressing parasites containing CD8 T cell epitopes could only stimulate CD8^+^ T cell when the LacZ protein was expressed in a PV-secreted form but not when expressed as a cytosolic form [20]. In addition, *T. gondii* expressing secreted OVA in the PV, but not those that express a cytosolic OVA, were able to stimulate OVA-specific CD4^+^ T cells [21]. Therefore, antigens secreted in the PV are more effective at activating both CD4 and CD8 T cell. PV-secreted antigens might be more efficiently released from infected cells and hence more antigen could be available to be processed through the MHC II pathway. Similarly, PV-secreted antigens are transported more effectively from the PV into the host cell cytoplasm possibly through cross-presentation dependent of the TAP transporter.

**Fig. 3:**
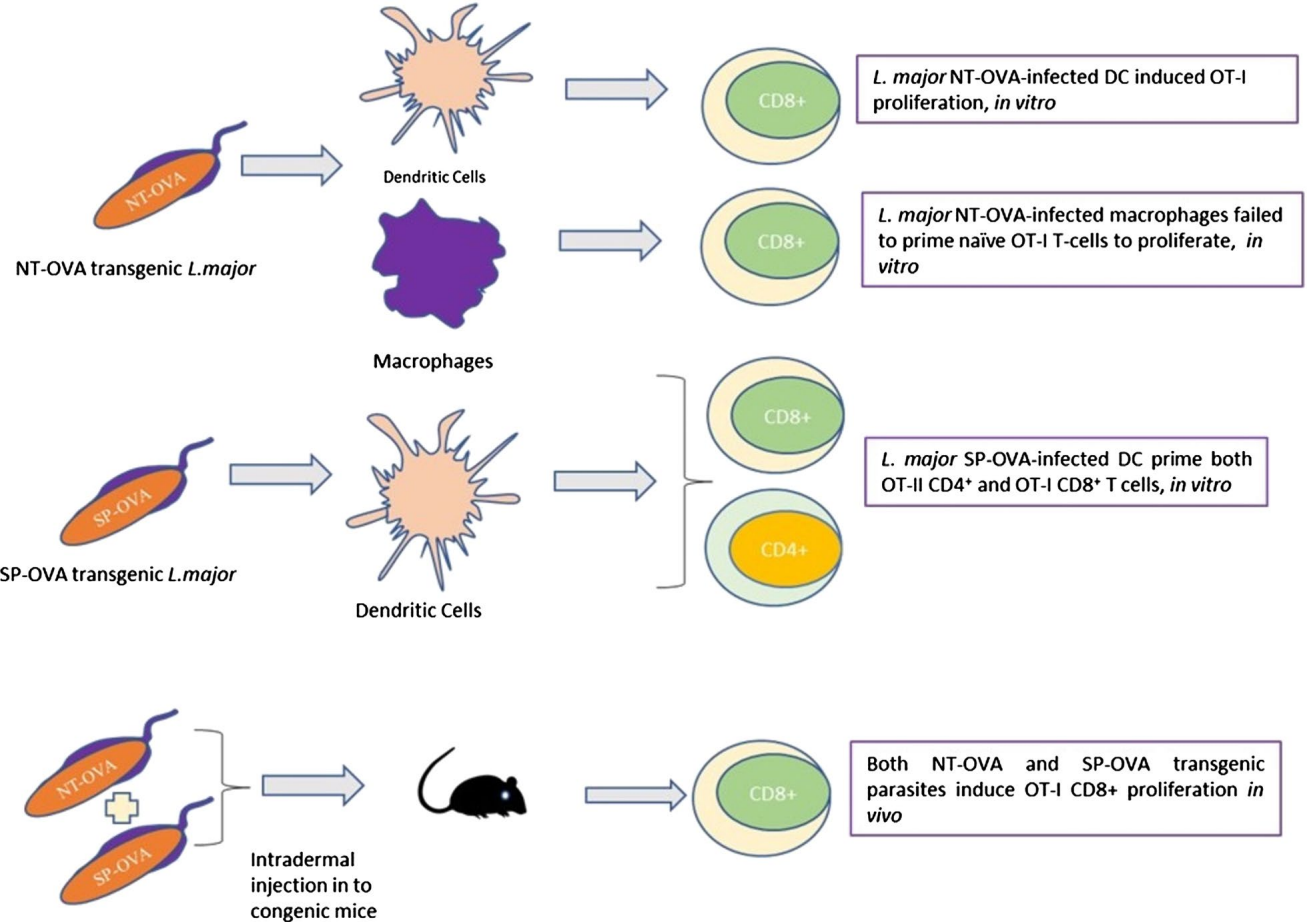
The expression of two OVA epitopes (NT-OVA and SP-OVA) in *L. major* parasite showed different type of T cell responses when exposed to dendritic cells and macrophages *in vitro* separately. Whereas, injection of both OVA epitope transgenic parasites into mice after adoptive transfer of OVA specific OT-I T cells showed only an induction of CD8^+^ T cells *in vivo*

Furthermore, *Leishmania* spp. have also been used to study the effect of expressed antigens at different locations on the induction of T cell responses. For example, OVA has been used as a reporter gene by integrating it into the genomic DNA of *Leishmania donovani*, allowing the isolation of OVA-expressing amastigotes. Mice adoptively transferred with OVA-specific OT-I T cells and infected with OVA-transgenic parasite demonstrated that OVA could be recognised in the context of MHC class I and in the same study a reduction in hepatic and splenic parasite burden was observed [22]. Furthermore, in a follow-up experiment, OVA expressed on the plasma membrane led to a significant activation of CD4^+^ T cells [23]. The ability of a fusion protein containing OVA (NT-OVA), expressed by transgenic parasites in either a secreted form or an intracellular form, to stimulate OT-I CD8^+^ T cells was investigated [24]. Dendritic cells infected by *Leishmania major* transfected with secreted NT-OVA could activate naïve OT-I cells while non-secreted (i.e. intracellular) NT-OVA was unable to achieve this outcome, possibly because less OVA was available in the phagosomes [24]. Indeed, high concentrations of NT-OVA might be required in the phagosomes, to allow for the relatively inefficient cross-presentation of OVA present in the phagosome (where the parasite resides), through the MHC I pathway. DCs pulsed with heat-killed parasites were also unable to activate naïve OT-I cells, possibly because not enough antigen can accumulate in the phagosomes and hence transit via cross-presentation to MHC I. Interestingly, infected macrophages, which generally lack the antigen cross-presentation pathway, were unable to activate naïve OT-I cells when infected with *L. major* transfected with either the secreted or the intracellular form of NT-OVA (Fig. 3), corroborating the results suggesting the use of cross-presentation in this process. Macrophages were able to activate primed OT-I cells but 20 times less efficiently, compared to DCs. Taken together, these results suggest that *L. major* antigens secreted in the phagosomes of DCs, but not macrophages, can cross-present antigenic peptides to MHC I leading to CD8^+^ T cells activation, while the local accumulation of intracellular antigens is insufficient to allow cross-presentation and hence no CD8 activation can occur. Interestingly, as noted above, similar conclusions were made for *T. gondii* except that in contrast to the *Leishmania*, *T. gondii* antigens in the PV do not seem to go directly into the MHC II antigen processing pathway.

### Expression of reporter genes for parasite tracking and immune cell characterization

The advent of model reporter gene technology has facilitated the understanding of many cellular responses to parasites with distinct phenotypic properties from the system being investigated.

Due to various limitations of light microscopy-based *in vivo* imaging techniques, the direct investigation of different events in the host has been impossible until recently [25]. One of the earliest studies of immune responses *in vivo* involved adoptive transfer of T cells, B cells, and DCs to a recipient host [26]. In the same context, different fluorescent dyes such as CFSE have been used for the labelling and short-term tracking of the cells as they migrate in the host. However, as cell division or activation results in dilution of the dye, tracking of the cell population over longer time periods was not possible in this model [25]. To overcome such difficulties, different fluorescent proteins have been expressed under promoters specific for various cell types such as, CD4^+^ T cells, CD8^+^ T cells, macrophages, DCs, and neutrophils and this has allowed long-term investigation of cell trafficking during *in vivo* immune responses [25]. This approach has been combined with the expression of different reporter genes in the parasite [27]. For example, transgenic *T. gondii* expressing luciferase enabled real-time monitoring of an infection *in vivo* [6]. In addition, several reporter molecules have been generated for cytokines such as IFN-γ, IL-2, IL-10 and IL-12 to understand how their production can be visualized and their association with host cells *in vivo* ascertained [28]. There has also been recent progress in using real-time imaging of host-pathogen interactions, for example such approach has been reported in *Toxoplasma*, *Plasmodium* and *Leishmania* [29].

*Toxoplasma gondii* expressing fluorescent markers have been used to distinguish infected macrophages, uninfected macrophages and DCs. Such transgenic parasites are also used to characterise the immunological phenotypes of these cells under flow cytometry [30, 31].

Another application for fluorescent marker-expressing parasites is the unravelling of the mechanisms leading to CM. For example, blood parasitemia and sequestration of parasites in the brain vasculature is the main predisposing factor for CM and both CD8^+^ and CD4^+^ T cells play important roles not only in the control of parasitemia, but also in the development of CM [32]. CD8^+^ T cells sequester in the brain tissue following *P. berghei* infection causing damage to the endothelial tissue of the brain through the production of perforins [33]. The association between Treg and CD8^+^ T cell recruitment in the pathogenesis of experimental CM was studied using *P. berghei* expressing luciferase and green fluorescent protein. Depletion of Treg cells with anti-CD25 monoclonal antibody leads to protection of mice from experimental cerebral malaria. In addition, the accumulation of parasites in the brain vasculature was reduced in these infected mice, resulting in a significant reduction of parasite burden. Mice lacking Treg cells showed higher numbers of activated CD4^+^ and CD8^+^ T cells in spleen and lymph nodes. However, CD8^+^ T cell recruitment to the brain was selectively reduced in these mice [34]. Thus, transgenic parasites have not only helped unravel the host’s immune response to wards the parasite but also the immune mechanisms contributing to a major aspect of the pathology of malaria.

Furthermore, a luciferase transgenic *L. donovani* has also been used to demonstrate a role for the Ras-related protein, Rab5, in the regulation of the phagosome-endosome fusion and how these subcellular compartments kill intracellular parasites [35]. Through expression of GFP in *L. major*, the temporal difference of the parasite antigens on the draining lymph node is demonstrated [36] and similar studies showed the role of lipophosphoglycan (LPG) in inducing DC-mediated pro-inflammatory responses using *Leishmania mexicana LPG1*^*−/−*^ mutants and this study reported high level of pro-inflammatory cytokine gene expression in the absence of LPG [37]. Therefore, the expression of reporter proteins by parasites has been important to track the events of parasite dissemination and replication during infection and such transgenesis technology has greatly increased the accessibility of improved humanized mouse models to investigate the protection efficacy of different parasite origin antigens, as discussed in the next section.

### Expression of antigens from other parasite species

**Fig. 4:**
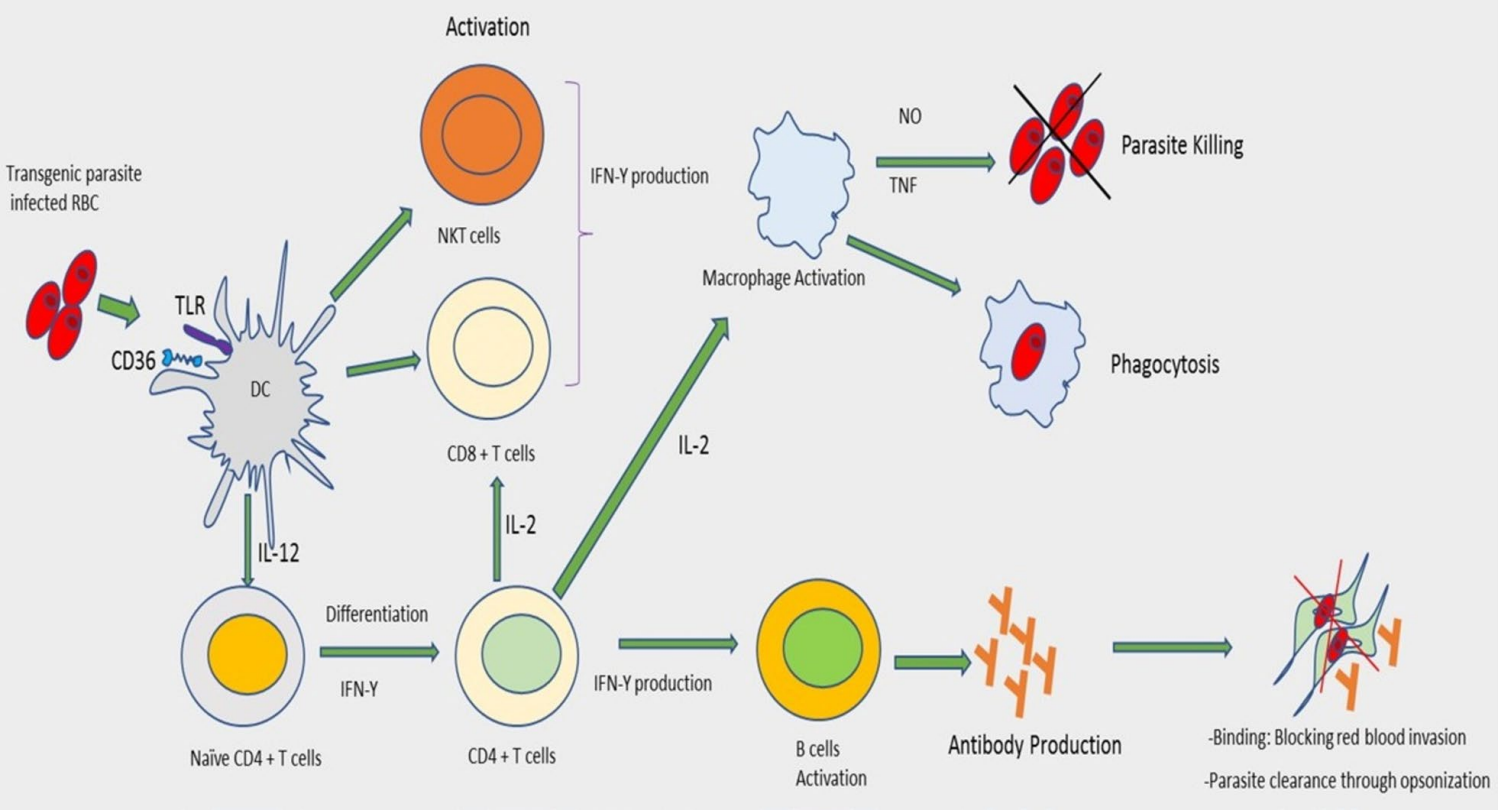
Transgenic *P. berghei* parasite expressing a circumsporozoite protein induces a strong antibody production and protection efficiency. Briefly, the transgenic malaria parasite lines infect the RBC of C57BL/6 mice and leads to the activation of B cells and IFN-γ production by CD4^+^ T cells. Passive transfer of antibodies to naïve recipient mice confers protection through opsonization process

In order to show that particular antigens are important targets for antibody-mediated protection, transgenic parasites have become a very useful tool (Fig. 4). For example antibodies play a significant role in the inhibition of blood stage merozoites by targeting the major protein component of MSP-1_19_ and therefore, by replacing the *P. berghei* allele with the *P. falciparum* one, MSP-1_19_ antibodies with a high level of inhibitory potential against the human parasite, were generated in mice. As a result, during challenge trials, a positive correlation between protection of mice from the blood stage malaria and the level of the surface protein expressed was observed. Moreover, full-length protein variants have been expressed and characterized in a transgenic *P. berghei* lines and patent infection of mice by these transgenic lines showed production of protective antibodies [38].

*Plasmodium berghei*, *P. chabaudi* and *P. yoelii* are three malaria species that infect mice and are most commonly used due to their shared pathological features with *P. falciparum*, the most pathogenic strains of human malaria parasite [39]. In order to investigate the potential of the circumsporozoite protein (CSP) as a vaccine antigen *in vivo*, the *csp* gene of rodent malaria parasites was replaced with the csp genes from human parasites such as *P. falciparum* and *P. vivax*. The chimeric rodent parasites could produce sporozoites in *Anopheles* stephensi mosquitoes, capable of infecting human and rodent hepatocytes [40]. Thus, by expressing recombinant antigens from a human parasite into a mouse parasite, it is possible to take advantage of a mouse model to identify neutralising immune responses in a mouse model and hence discover potential vaccine candidates for human malaria.

### Expression of transgenes by parasites to improve their immunogenicity

**Fig. 5:**
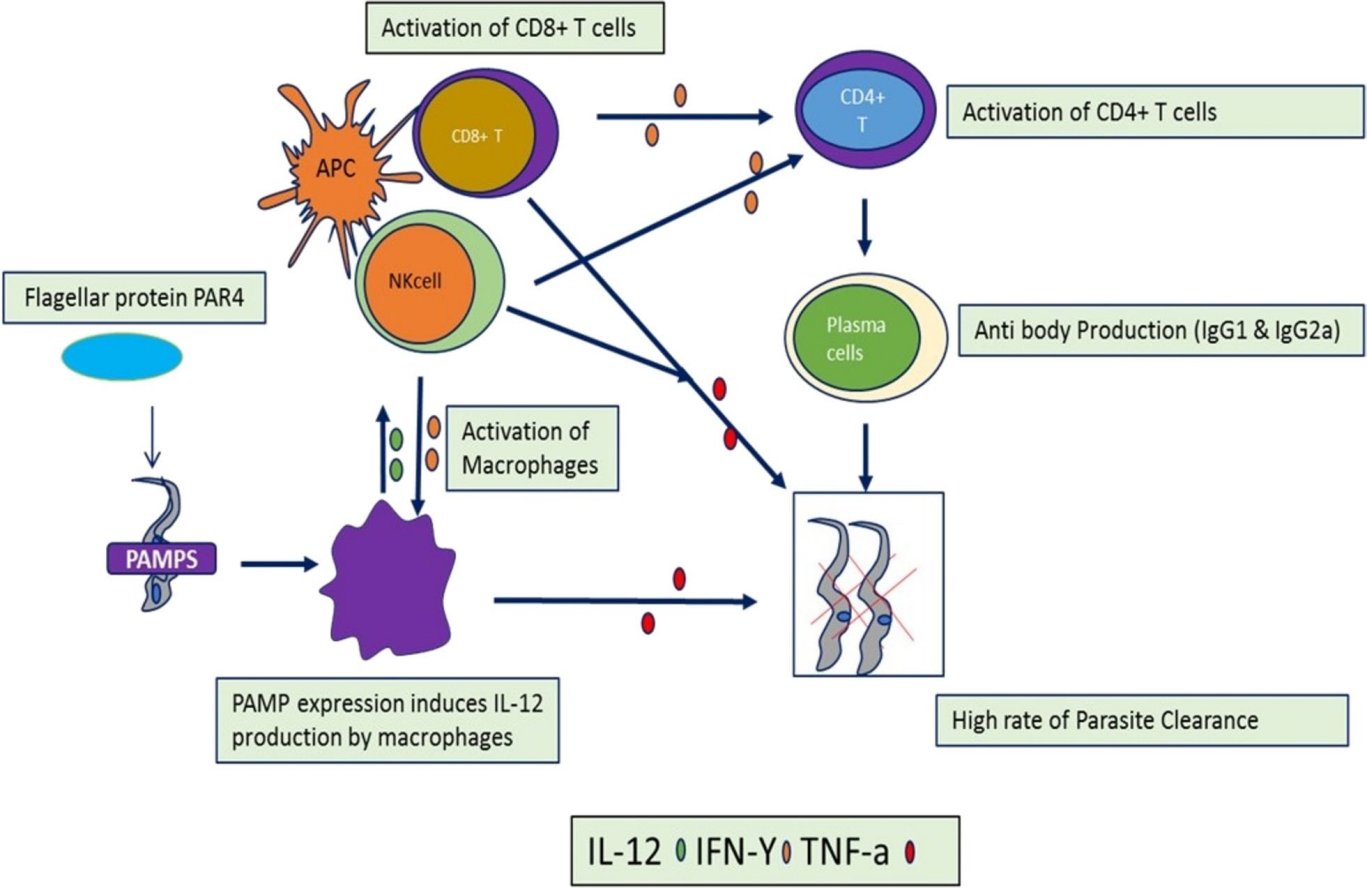
Expression of flagellar protein PAR4 in *T. cruzi* causes the activation of macrophages, CD8^+^ T cells and NKT cells which leads to the high rate of parasite destruction from the circulation through the production of different cytokines having direct effect such as TNF-a and through the activation of plasma cells and production of protective antibodies

Many parasites use immunomodulatory mechanisms to ensure their survival in the host by crippling the host’s immune response, most often through the production of immunomodulatory molecules [41], which bind or interact with host immune cells [42]. Others have developed ways of remaining silent by virtue of low or absent expression of pathogen associated molecular patterns (PAMPs). Thus, when parasites evade the immune system by suppressing expression of PAMPs, it might be possible to express PAMPs transgenes from other organisms, and hence attract the attention of the immune system to the parasite, making them more immunogenic. To unravel this issue, transgenic *T. cruzi* expressing an exogenous PAMP with the ability to activate the innate immune system such as (i) *Salmonella typhimurium* flagellin (FliC), (ii) *Neisseria meningitidis* (FAM18 strain) porin (PorB) or (iii) *T. cruzi* paraflagellar rod protein 4 (PAR4) genes have been generated. Not surprisingly, strong innate immune responses were observed when mice were infected with these transgenic parasites and the PAMPs activated innate immune cells and these activated immune cells played an important role in controlling *T. cruzi* infection [43]. These results suggest that a relative deficiency of PAMPs in *T. cruzi* helps the pathogen to survive in the host. These studies also showed that inoculation of exogenous PAMPs with *T. cruzi* and under continuous expression can induce strong CD8^+^ T cell responses which ultimately leads to the control and clearance of infection (Fig. 5). A similar study aimed at optimal immune control and clearance of parasites persisted in the host also showed that transgenic *T. cruzi* expressing flagellar protein PAR4 was more effective at inducing PAR4-specific CD8^+^ T cell responses, which improved protection during subsequent parasite challenges [43]. Hence, these experiments demonstrate that the relative absence of PAMPs in *T. cruzi* can be harnessed to study the importance of PAMPs from other sources, in the induction of adaptive immunity.

The two functionally distinct T helper cell populations, which determine the *Leishmania* infections are the Th1 cells known for secretion of IL-2 and IFN-γ cytokines and Th2 cells known for the secretions of IL-4, IL-10 and IL-13. The susceptibility to *Leishmania* infection is associated with Th2 proliferation and secretion of their cytokine signatures mentioned above and resistance to infection is maintained by the secretion IFN-γ by CD4^+^ Th1 helper cells [44]. However, there is no clear evidence how the parasite skewed the immune response and leads into protection and disease progression. *L. major* secreting the mouse cytokine granulocyte-macrophage colony-stimulating factor (GM-CSF) is engineered to induce protective host immune responses [45]. Indeed, GM-CSF induces secretion of many pro-inflammatory cytokines such as IL-1β, IL-18 and IL-6 by macrophages and, as a result, transgenic parasites expressing GM-CSF failed to survive. Transgenic *Leishmania* spp. engineered to produce monocyte chemoattractant protein 1 (MCP-1) were less pathogenic in susceptible BALB/c mice *in vivo*, with significant reduction in lesion size compared to wild-type parasites [46]. In addition, parasites producing MCP-1 caused CCR2 positive macrophage recruitment, but the innate immune response was less efficient at inducing protective immunity against subsequent challenges.

In another application, transgenic parasites can also be used as vaccine vehicles. Indeed, one study showed the development of a novel system for the expression of nematode secreted proteins in another parasite such as Trypanosoma musculi, a natural parasite of mice. Furthermore, acetylcholinesterase from *Nippostrongylus brasiliensis* was engineered in trypanosomes and these transgenic trypanosomes were able to induce a protective immune response against *N. brasiliensis*, by altering the cytokine environment. In the same study, immune cells exposed to acetylcholinesterase expressing parasites *in vivo* showed activation of macrophages, production of high levels of nitric oxide and decreasing arginase activity [47].

In summary, the transgenesis of proteins that improve immunogenicity of the parasite have given us insights not only in how parasites evade the immune system, for example by reducing the amount of danger signals they express but more importantly also in the fundamental mechanisms of immune regulation, particularly the role of key cytokines. Some of these engineered parasites might even be used as vaccine vehicles, although this would require passing considerable regulatory hurdles.

### Deleting and knocking-down parasite genes to evaluate their effect on immune responses

Deletion mutants of parasites have been used to investigate the role of critical molecules in the binding of the parasite to the host cells and the effect of antibodies to these molecules in inhibiting this process. Indeed, studies in human malaria showed that erythrocyte binding-like (EBL) proteins and reticulocyte binding-like (RBL or PfRh) proteins are involved in erythrocyte bindings and are targets of human invasion inhibitory antibodies in addition to being important components of acquired protective immunity [48]. A construct of *P. falciparum* lines in which *eba-175*, *eba-181*, and *eba-140* genes were knocked out by targeted disruption, showed that the EBL and PfRh proteins functionally interact during merozoite invasion. Further evidence showed that PfRh and EBL proteins induced antibodies that potently blocked merozoite invasions and hence these antigens could potentially be used as vaccine candidates [48, 49]. The targeted disruption of key genes to establish a role for antibodies to specific proteins can only be used when knock-out parasites are viable, as is the case with the *eba* genes. However, in some instances such as the MSP-1 protein a knock-out strategy can be unsuccessful [50], for example if the protein is essential for parasite survival. In such cases researchers have resorted to the more complex approach of expressing the protein from one species of malaria into another (see below), to demonstrate the role of antibodies in protection. Another alternative is the use of RNAi, which by its nature downregulates the expression of the protein but does not abolish expression altogether.

The ability of parasites to modulate immune responses is often mediated by the production of immunomodulatory molecules. Hence, there is a growing interest in the characterization of the mediators released by the parasites and analysing how such molecules can redirect the host’s immune response [42]. This might also have a tremendous advantage in developing unique and targeted therapies not only against the parasites [51], but also potentially for the treatment of other immune-mediated conditions.

**Fig. 6:**
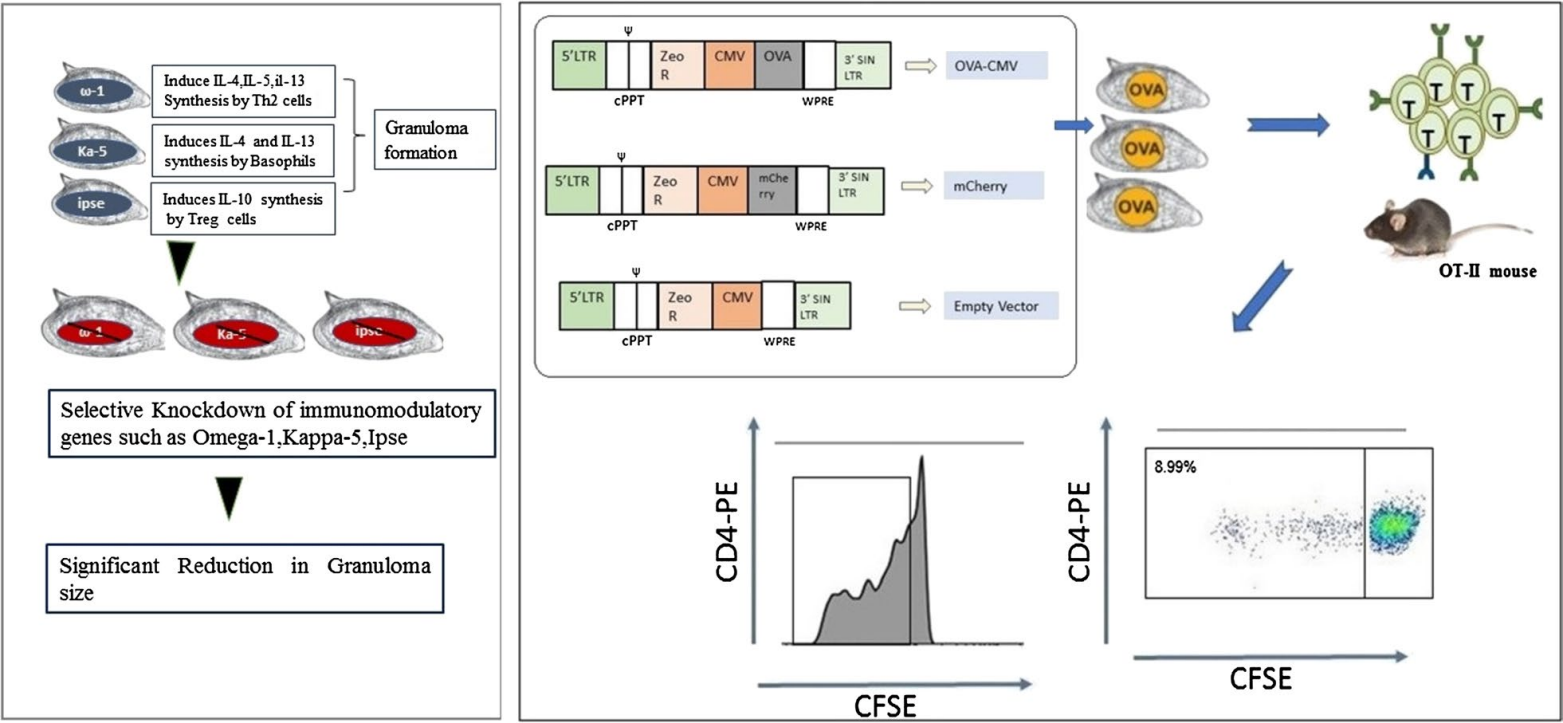
*Schistosoma mansoni* eggs transduced with lentiviruses containing shRNAmir showed a significant reduction the size of granuloma comparing with the untraduced eggs [8] and the expression of chicken ovalbumin in *S. mansoni* eggs after delivery of the OVA transgene through the lentiviral transduction system leads in to the recognition of the OVA by OT-II T cells *in vitro*

Parasites, such as *Schistosoma mansoni*, are among the pathogenic organisms that invade diverse organ systems such as the lymphatic system, the gastro-intestinal tract and the vascular system with multiple immunomodulatory lines of attack on the immune system. Co-evolution of parasites and their hosts’ immune system has resulted in a delicate balance that allows chronic infections to be maintained without provoking fatal immunopathology or overwhelming infection in the host [52, 53]. *S. mansoni* egg proteins including Omega-1, IPSE, and kappa-5 are the main contributors of egg-induced immune regulation [54]. The effect of soluble egg antigens (SEA), such as *Omega-1*, is attributed to an altered interaction of DCs with Th cells and the initiation of mRNA and rRNA degradation. This leads to abolished protein-expression by DCs. These interactions and the immunomodulatory mechanisms can be investigated by downregulating Omega-1 in *S. mansoni* eggs and studying the effect *in vivo* [8]. Omega-1 alone can induce a Th2 response *in vitro* and *in vivo* through the conditioning of DCs. Indeed, the depletion of this protein totally abrogates the maturation of T cells induced by schistosome SEA *in vitro* [55]. As mentioned in the introduction, multicellular parasites are difficult to transfect using classical molecular techniques and of the require lentivirus-mediated transgenesis. Lentiviral transduction system mediated knocking-down of Omega-1 in *S. mansoni* eggs leads to the production of IFN-γ [8] (Fig. 6). In the same study, lung cell suspensions from naïve mice, which were intravenously injected with wild-type and empty vector transduced *S. mansoni* eggs revealed a characteristic Th2 cytokine profile (IL-4, IL-5, IL-6, IL-10 & IL-13) with low levels of Th1 (IL-1α, IL-12, INF-γ, TNF-α), and Th17 cytokines (IL-17, IL-22). Moreover, intravenous injection of wild-type *S. mansoni* eggs increased numbers of eosinophils, granulocytes, T helper cells, alveolar APCs and B cells [8]. In contrast, mice injected with Omega-1 knock-down eggs had a cytokine profile characterised by a slight increase in Th1 cytokines, such as TNF-α. However, no significant difference in the levels of Th1, Th2, and Th17 cytokines was observed between the Omega-1 knock-down eggs and wild-type or the empty vector controls. However, Omega-1 knock-down parasites had reduced pathology in the lungs, suggesting an effect on granuloma formation. Although Omega-1 is the principal component of SEA, which conditions DCs for priming Th2 responses, it is not the only glycoprotein in SEA that can induce these responses. Other egg proteins, such as IPSE and Kappa-5 also play a role in immunomodulation of the host’s immune response. Hence, knock-down of Ipse and Kappa-5 leads to a slight decrease in IL-2 and IL-10 production and an increase in secretion of IL-1 and IL-6 but, the overall difference in the level of secreted cytokines was not significant for either of the SEA knock-down groups.

In recent years the CRISPR/Cas9-based genome edition tools have become a standard way of disrupting key genes in order to study their effect. However, most of the studies so far have used this technique to investigate the parasite biology rather than host-parasite interactions, which fall outside of the scope of this study. While one can expect many future studies to include CRISPR/Cas9-based genome edition tools in the study of immune responses to parasite, the only study so far targeted Omega-1 of *S. mansoni* eggs and the results conformed these discovered by downregulating this gene using the RNAi/lentiviral transduction system, namely a reduction of pulmonary granuloma [56].

In another parasite example, deletion of *L. mexicana* cysteine peptidase B in these parasites resulted in similar lesion-development compared to wild-type parasites in the early stages of infections [57]. However, mice infected with CPB^-/-^ parasites cleared the infection after inducing a STAT4 and IL-12-dependent Th1 response, indicating that cysteine peptidase B suppresses the immune response [58].

Finally, disruption of the conserved *6-Cys* B gene family, which plays a role during development of sexual stages of *Babesia bovis,* resulted in inhibiting parasite invasion of red blood cells [59]. Such novel gene manipulation approach could generate attenuated parasites usable as vaccines against babesiosis.

Taken together these experiments demonstrate the usefulness of different gene-disruption and downregulation technologies for the study of gene function of parasites including these highlighted here in relation to host-parasite interactions. While gene disruption offers a more absolute effect because the protein of interest is completely absent, gene downregulation has the advantage that if the gene is essential the parasite might survive although in an impaired state and hence an effect might still be observable in that case.

### Summary and future directions

As outlined in this review, recent advances in transgenesis of complex parasites have allowed significant progress towards understanding the immunobiology of a range of parasites. These advances could potentially also be applied to even larger parasites, such as parasitic nematodes. Indeed, the tracking of nematode-specific T cell responses has so far been a challenge [60]. The use of nematodes expressing model antigens, such as OVA, might help in this respect, particularly if combined with T cell receptor transgenic mice. Studies over the last two decades involving *in vivo* depletion of specific cells, cytokines and their receptors showed that the immune response to nematodes in a murine model is mainly dependent CD4^+^ T cells [61, 62]. Parasitic nematodes induce immunomodulatory mechanisms to invade their host and sustain their infectivity [63]. While there is a general mechanism of immunomodulation by nematodes which involves the release of molecules capable of manipulating host immune responses, the nature, and mechanisms of altering the host’s immunity is different from one parasitic nematode to the other [41]. Therefore, to help with this endeavour knock-down or deletion of specific genes suspected to have effects on the immune system can be generated and used to confirm their respective roles.

Importantly, parasites in which one key immune mechanism has been eliminated could potentially reveal additional mechanisms that are normally masked by the overpowering effect of the main immune mechanism. The use of transgenic parasites in studying immune response is summarised in Table 1. Therefore, we forwarded the following learning points.

**Table 1:** Summary of a list of different transgenic parasites and gene constructs with corresponding functions

Genus	Species	Gene construct/transgenic parasite	Study performed or Function	Reference
*Plasmodium*	*P. berghei*	*P. berghei* PbGAG/ HIV-1 Gag	Expression of HIV-1 Gag in the transgenic blood stage parasites and demonstrating its role in the protection against vaccinia virus-gag and malarial parasites	[64]
		*P. berghei*-Fluspo	Examination of the motility and movement of sporozoites in the salivary gland of mosquito during host infection	[65, 66]
		*P. berghei*-GFP-luciferase	Tracking of parasite sequestration during erythrocyte development and identifying the role of CD36 and Tregs in ECM development and immunity	[34, 67]
		*P. berghei* expressing full-length PvCSP (VK247)	The *P. berghei* expressing full-length PvCSP (VK247) was generated and examined its applicability to CSP based vaccine trial by examining its biological characteristics in mosquitoes and mice	[68]
	*P. falciparum*	*P. berghei* MSP-119	The transfected parasite line and parental parasites that differ only in MSP-119 were compared and antibodies specific for this domain are a major component of the inhibitory response in *P. falciparum* immune humans and *P. chabaudi* immune mice	[69]
	*P. yoelii*	*P. yoelii*-GFPCON (EF1α-promoter)	Quantification, characterization, and imaging of malaria parasites in the liver *in vivo*	[70]
		*P. yoelii* p52–/p36–	Genetic manipulation of p52/p36 gene and its role in the development of infection and protective immunity	[71]
	*P. vivax*	*P. falciparum* and *P. berghei* lines expressing PvDHFR-TS	Expression of Pv DHFR-TS and performing anti-malarial drug screening assay and drug sensitivity in both transgenic and non-transgenic parasite models	[72]
	*P. knowlesi*	*P. knowlesi* expressing bioactive host gamma interferon	Transgenic *P. knowlesi* parasite was generated to express rhIFNg and using live attenuated whole organism vaccine, how parasite-host interactions was evaluated	[73]
*Trypanosoma*	*T. cruzi*	ADC transgenic *T. cruzi*	Modulation of oat arginine decarboxylase gene expression and genome organization in transgenic *T. cruzi* epimastigotes	[74]
*Leishmania*	*L. major*	OVA transgenic *L. major*	Expression of truncated OVA and *in vivo* recognition of the parasites by OVA-specific T cells	[23, 24]
	*L. major*	*L. major* MPK10	Using the transgenic approach, the regulation of MAP Kinase MPK10 reveals an auto-inhibitory mechanism, which is important for the parasite stage specific regulation and parasite viability	[75]
	*L. chagasi*	rRNA promoter regions	Characterization of the gene after transfected into parasite and analysing its function in promoting the expression of proteins from *Leishmania* plasmid	[76]
	*L. tarentolae*	Human tissue-type plasminogen activator expression	Transfection of *L. tarentolae* with expression vector containing tPA gene and its functional analysis. The expression cassette including tPA gene integrated in *Leishmania 18S* rRNA genes through homologous recombination and transfected *Leishmania* produce biologically active tPA	[77]
	*L. denovoni*	LdCen−/− or Ldp27−/− parasites	Role of pro-inflammatory cytokine IL-17 in *Leishmania* pathogenesis and in protective immunity by *Leishmania* vaccines	[78]
	*L. mexicana*	*L. mexicana LPG1−/−*	Transgenic episomal expression of a reporter antigen, *E. coli* β-galactosidase (β-gal), to study the mechanisms that result in the cross-presentation of exogenous antigens to the Class I Pathway for the stimulation of CD8^+^ T cells	[79]
*Schistosoma*	*S. mansoni*	Transfection of RNAi cloned with pGIPZ expression vector into parasite egg	Omega-1 knock-down in *S. mansoni* eggs by lentivirus transduction reduces granuloma size *in vivo*	[8]
*Toxoplasma*	*T. gondii*	Mitochondrial association factor 1 (MAF1).	Exogenous expression of MAF1 to show that it binds host mitochondria and thus MAF1 is the parasite protein directly responsible for HMA. The association with host mitochondria may represent a novel means by which *Toxoplasma* tachyzoites manipulate the host	[80]
	*T. gondii*	*T. gondii* -YFP	Yellow fluorescent protein (YFP) was transfected in *T. gondii* RH strain in the cytoplasm. The transgenic protein was injected to chickens SC and provide protection against *E. tenella* YFP infection. *T. gondii* YFP induced low levels of antibodies to YFP in chickens, suggesting that YFP specific cellular immune response was probably responsible for the protective immunity against *E. tenella* YFP infection	[81]
	*T. gondii*	TCR/Kb/OVA_257–264_	Using transgenic β 2M^(−*I*−)^ mice, several peptides with the ability to induce positive selection were identified	[82]
	*T. gondii*	TCR/H-2b/OVA_323–339_	Generation of MHC class II-restricted, OVA-specific αβ TCR transgenic mice and analysing the recognition of OVA_323–339_ by mature OT-II T cells	[83]
	*T. gondii*	I-Ab-EGFP knock-in (H–2b)	The mechanisms how the class II molecule transport in live in live APCs by replacing the mouse MHC class II gene with a version that codes for a class II molecule tagged with enhanced green fluorescent protein (EGFP)	[84]
	*T. gondii*	GRA6-derived HF10 epitope transgenic *T. gondi*	Location of the CD8^+^ T cell epitope within the antigenic precursor determines immunogenicity and protection against the *T. gondii* parasite	[85]
*Eimeria*	*E. tenella*	*E. tenella*-CjaA with mCitrine reporter	Comparison of the result in the protective immunity of the vaccination of specific pathogen free chickens by single or multiple oral inoculation of *E. tenella*-CjaA oocysts with unvaccinated and wild-type *E. tenella* vaccinated controls	[86]
	*E. mitis*	Sporozoites of *E. mitis* transfected with enhanced yellow fluorescent protein (EYFP)	After expression of EYFP in transgenic *E. mitis*, the peripheral location of the nuclei of mature microgametocytes in microgametogenesis revealed that microgametes were directly differentiated along with the migrating of nuclei to peripheral location	[87]

- We have reviewed current advances focusing on the use of transgenesis as a tool in the study of host-parasite interactions.
- The transgenesis approach is now becoming a novel technique in the integration of foreign genes reliably into the host genome for functional analysis of the immune response *in vivo*.
- To date, few transgenic parasites have been developed and improvements in the advancement of this field is required to increase our knowledge of parasite immunology.
- We foresee that such advancement in transgenesis technology could pave the way to rapid developments in functional genomics as it relates to host-parasite interactions. For example, high throughput insertional mutagenesis, expansion of RNAi for *in vivo* studies might soon become a practical reality.
- We also expect the advances achieved thus far can be adapted in a wide range of pathogenic parasites to understand their nature of interaction with the host.

## Conclusions

In conclusion, transgenic parasites have been utilised in a wide range of applications to investigate complex host-parasite interactions *in vivo*. Indeed, using this versatile technology, it is now possible to unravel the detailed immune response to parasites. While this review highlights how transgenic parasites have influenced our understanding of the host-parasite interaction, many additional questions remain and establishing these advanced transgenesis methods for applications in an ever-increasing variety of parasite species will undoubtedly result in the discovery of fascinating insights about the interactions of parasites with the immune system of their hosts.

## Data Availability

Not applicable.

## Abbreviations

RNAi: RNA interference
TCR: T cell receptor
MHC-I: major histo compartment I
MHC-II: major histo compartment II
DCs: dendritic cells
CM: cerebral malaria
PVM: parasitophorous vacuole membrane
LPG: lipophosphoglycan
OVA: chicken ovalbumin
CSP: circumsporozoite protein
MSP-_19_: merozoite surface protein
PAMPs: pathogen-associated molecular pattern
SEA: soluble egg antigens
mRNA: messenger RNA
rRNA: ribosomal RNA
GM-CSE: granulocyte-macrophage colony-stimulating factor
